# Target Uncertainty During Motor Decision-Making: The Time Course of Movement Variability Reveals the Effect of Different Sources of Uncertainty on the Control of Reaching Movements

**DOI:** 10.3389/fpsyg.2019.00041

**Published:** 2019-01-28

**Authors:** Melanie Krüger, Joachim Hermsdörfer

**Affiliations:** Chair of Human Movement Science, TUM Department of Sport and Health Sciences, Technical University of Munich, Munich, Germany

**Keywords:** reaching movements, sensorimotor control, movement planning, motor control, embodied decision making, time course of variability, kinematics

## Abstract

The processes underlying motor decision-making have recently caught considerable amount of scientific attention, focusing on the integration of empirical evidence from sensorimotor control research with psychological theories and computational models on decision-making. Empirical studies on motor decision-making suggest that the kinematics of goal-directed reaching movements are sensitive to the level of target uncertainty during movement planning. However, the source of uncertainty as a relevant factor influencing the process of motor decision-making has not been sufficiently considered, yet. In this study, we test the assumption that the source of target uncertainty has an effect on motor decision-making, which can be proven by analyzing movement variability during the time course of movement execution. Ten healthy young adults performed three blocks with 66 trials of goal-directed reaching movements in each block, across which the source and level of reach target uncertainty at movement onset were manipulated (“no uncertainty”, “extrinsic uncertainty”, and “intrinsic uncertainty”). Fingertip position of the right index finger was recorded using an optical motion tracking system. Standard kinematic measures (i.e., path length and movement duration) as well as variability of fingertip position across the time course of movement execution and at movement end were analyzed. In line with previous studies, we found that a high level of extrinsic target uncertainty leads to increased overall movement duration, which could be attributed to increased path length in this condition, as compared to intrinsic and no target uncertainty (all *p* < 0.001). Movement duration and path length did not show any differences between the latter two conditions. However, the time course analysis of movement variability revealed significant differences between these two conditions, with increased variability of fingertip position in the presence of intrinsic target uncertainty (Condition × Sampling point: *p* = 0.01), though considerably less than under high extrinsic target uncertainty (*p* ≤ 0.001). These findings suggest that both the level and source of uncertainty have a significant effect on the processing of potential action plans during motor decision-making, which can be revealed through the analysis of the time course of movement variability at the end-effector level.

## Introduction

In everyday life, we are constantly forced to make decisions, often under dynamic and uncertain conditions. This encompasses simple, practical decision, such as whether to take along an umbrella to protect oneself from the potential rain in the afternoon, as well as complex, more abstract decisions, e.g., how the invest the savings to maximize return in 20 years from now. While in some fields of research, e.g., psychology and economics, decision making has a long scientific history (Edwards, 1954; Kahneman and Egan, 2011), motor decision making has more recently caught scientific interest (see e.g., Wu et al., 2015; Gallivan et al., 2018). In this context, motor decision-making can be broadly defined as the process of choosing an action plan from a range of multiple potential actions (Wolpert and Landy, 2012; Wu et al., 2015). Movement planning (often mainly referring to the process of action specification) has been widely investigated for different motor tasks and populations in motor control research. However, the integration of this work with computational models and psychological theories of decision-making has only recently begun (e.g., Trommershäuser et al., 2008; Song and Nakayama, 2009; Ramakrishnan and Murthy, 2013).

Traditionally, movement planning has been assumed to consist of serially organized processes. This includes the selection of the required action to achieve the movement goal, followed by the specification of this action, and finally the issuing of the respective motor command for action execution. Whether perceptual decision making on the movement goal should also be considered as part of the movement planning processes or not is still under debate (Wong et al., 2015) and may depend on the precise definition of motor decision-making. While the theory of serially organized movement planning processes seems to be able to explain a wide range of observable movement patterns, it is not well able to describe rapid changes in movement execution that might be necessary in the presence of dynamic environmental conditions. In addition, recent neurophysiological studies found simultaneous activity in different brain areas assumed to be involved in either action selection or specification in humans and non-human primates (Cisek and Kalaska, 2010; Petzschner and Krüger, 2012). As a theoretical explanation of these findings, the affordance competition hypothesis was proposed (Cisek, 2007). The key assumption of this theory is the parallelism of action selection and specification processes to account for the dynamics and uncertainties in the environment during movement planning. However, the human motor system not only has to account for uncertainties and environmental dynamics during movement planning, but also during movement execution. In order to reflect this point, Cisek and Pastor-Bernier (2014) postulated the theory of embodied decision making. Following this theory, action selection and specification run in parallel not only until movement initiation, but are ongoing processes even during movement execution. This would allow for flexibly changing movement plans during the course of movement execution. In line with this assumption, Gallivan and colleagues provided empirical evidence for the competition of multiple potential action plans even after movement onset using kinematic movement analysis (e.g., Gallivan and Chapman, 2014; Nashed et al., 2017; Gallivan et al., 2018). These studies tested their assumptions using a research paradigm in which participants had to perform rapid reach movements under target uncertainty.

Uncertainty is a central term in (motor) decision-making research. Critically, Downey and Slocum (1975) noted already more than 40 years ago that this term is commonly used without further definition, in the assumption that everybody knows what it means. A study by Lipshitz and Strauss (1997) revealed the many different conceptualizations of uncertainty in the literature, including for example the equalization of uncertainty with risk or ambiguity. Based on this, they propose the classification of uncertainty either according to its issue or according to the source of uncertainty. In that context, they identify three basic sources of uncertainty: incomplete information, inadequate understanding or undifferentiated alternatives. Following the logic of Lipshitz and Strauss (1997), incomplete information refers to the complete lack or only partial knowledge about the (probability of) occurrence of events and their consequences. It is often also referred to as “risk” in the literature (e.g., Hsu et al., 2005). Lipshitz and Strauss (1997) further mention it to be the most commonly cited source of uncertainty. This might be explained by the fact that this source of uncertainty is experimentally or externally well controllable. Inadequate information, on the other hand, refers to the inability to decide on actions because of the lack of understanding of the available information and their consequences. To put it simply, individuals who are uncertain about their decision due to inadequate understanding just do not know what to do with the available information. Last, undifferentiated alternatives correspond to the source of uncertainty that arises from the presence of equally attractive choice option, given that all relevant information are available and fully understood. It is also sometimes referred to as “conflict” in the literature [see Lipshitz and Strauss (1997) for an overview about synonyms used in the literature for the different sources of uncertainty].

With this differentiation in mind, a closer look on the manipulation of uncertainty in studies on motor decision-making in reaching movements seems appropriate. Generally, two different choice conditions can be found in the literature – forced choice and free choice. In forced choice conditions, which draw on the externally imposed (in-)completeness of target information as the source of uncertainty, participants are cued to rapidly reach towards one of multiple potential reach targets. This cue can appear either before or after movement onset [termed “go-after-you know” or “go-before-you-know” tasks, respectively, Gallivan et al. (2018)]. While in the first version of this condition, the level of target uncertainty is minimal, since the participants have complete information of the reach target before movement onset, the level of target uncertainty is high in the second version. In general, the less predictable the cue on the final reach target is or the later it appears, the greater is the extrinsic uncertainty during movement planning. In contrast, the free choice condition draws on ambiguity (“undifferentiated alternatives”) as source of uncertainty, which originates from an intrinsic indecision about choice options. In this condition, individuals have to process and weigh available information and, based on the outcome, freely choose between multiple potential actions. Thus, intrinsic uncertainty does not arise from an externally controlled incompleteness of available information that are required to decide on an action plan, but from an intrinsic limitation to decide for one action plan against another in the presence of all relevant information. Consequently, the more similar potential actions are (e.g., in their costs or likelihood of success), the greater is the intrinsic uncertainty about which motor action to decide on in free choice-tasks. While in the studies reported above (Gallivan and Chapman, 2014; Nashed et al., 2017; Gallivan et al., 2018) the “go-after-you-know”- and free choice-tasks are commonly used as control conditions for the “go-before-you-know”-task, the different source of uncertainty (extrinsic vs. intrinsic) in these conditions is not made explicit.

However, this distinction becomes of fundamental relevance when considering the implications of different sources of uncertainty for motor decision-making strategies. While experimental set-ups using a “go-before-you-know”-task, i.e., causing “extrinsic uncertainty”, enforce the parallel processing of multiple potential action plans even after movement onset (at least up until the final reach target is cued), free choice conditions, inducing “intrinsic uncertainty”, principally allow a serial order of action selection-specification and action execution processes, similar to the “go-after-you-know”-task. While the parallel preparation of multiple potential action plans might be beneficial to cope with uncertainties and environmental dynamics during movement execution, a serial processing strategy is beneficial for minimizing target uncertainty at movement onset. Thus, when individuals can freely choose between ambiguous movement targets, a strategy to reduce uncertainty at movement onset would be to decide for one of the potential action plans immediately after stimulus onset and executing the movement with a minimum of reach target uncertainty. The question of whether and how different sources of uncertainty (extrinsic vs. intrinsic) affect the parallel processing of multiple potential action plans during motor decision-making still needs to be investigated and is addressed in this study.

A promising methodological approach to reveal the differences in motor decision-making related to different sources of uncertainty is to analyze movement variability during the time course of movement execution. The analysis of endpoint variability as a kinematic measure of task performance is well established in motor control research, at least since Fitts’ seminal work on the relationship between movement speed and accuracy (Fitts, 1954; Fitts and Peterson, 1964). Ample empirical evidence suggests that endpoint variability is generally low in healthy young and older adults, but sensitive to different environmental and task constraints (e.g., Gordon et al., 1994; Desmurget et al., 1997; Faisal and Wolpert, 2009; Krüger et al., 2011). The time course of movement variability is supposed to contain additional relevant information about the process through which the underlying motor control strategies come into effect (e.g., Morishige et al., 2006; Krüger et al., 2011; Krüger et al., 2012; Verrel et al., 2012; Krüger et al., 2013). Important for the context of this study, changes in the time course of movement variability at the effector level (e.g., joint angles of the arm) have previously been explained as adjustments of the sensorimotor system to uncertain planning conditions (de Freitas et al., 2007). Empirical evidence has accumulated suggesting that these adjustments become evident as changes in the coordination of the naturally abundant effector degrees of freedom (DoF). In effect, variability in task-relevant directions is minimized, by simultaneously allowing flexibility (i.e., variability) in task-irrelevant directions (e.g., Scholz and Schöner, 1999; Latash et al., 2002; Müller and Sternad, 2004; Liu and Todorov, 2007; Gera et al., 2010; Krüger et al., 2012). This assumption is supported by motor control theories, e.g., optimal feedback control: Todorov and Jordan (2002); Todorov (2004); also see Harris and Wolpert (1998).

In sum, recent scientific efforts have established a link between motor decision-making and sensorimotor control. Empirical evidence suggests a parallelism of action selection and specification to account for uncertainties during movement planning (Chapman et al., 2010; Gallivan and Chapman, 2014). Further, theories hypothesize multiple action planning as an ongoing process even during movement execution to cope with the dynamics in the environment (Cisek and Pastor-Bernier, 2014). These assumptions were supported by empirical evidence highlighting differences in movement kinematics (e.g., movement duration and path length) between reaching movements with or without target uncertainty. However, the source of target uncertainty as a relevant factor influencing the competition of multiple potential action plans during motor decision-making has not been sufficiently considered, yet. In this study, we test the assumption that the source of target uncertainty has an effect on the parallel processing of multiple potential action plans during motor decision-making, which can be proven by analyzing movement variability during the time course of movement execution. On that account, we performed an experiment where participants had to reach towards circular targets for which we varied the sources and levels of target uncertainty. Besides kinematic measures, which can be standardly found in studies on motor decision-making [e.g., path length and movement duration, Gallivan and Chapman (2014)] we analyzed the time course of variability of the fingertip position to gain additional insight into to underlying motor control strategies to cope with uncertainties during motor decision-making.

## Methods

### Participants

Ten healthy adults (six female, mean age ± SD: 29.3 ± 4.1 years) voluntarily participated in this study. All were dominantly right handed, as assessed by means of the Oldfield Handedness Inventory (Oldfield, 1971), had normal or corrected-to-normal vision, no neurological impairment and gave written informed consent before participating in the study. The study protocol was in accordance with the Declaration of Helsinki and approved by the Ethical committee of the Medical Faculty, Technical University of Munich.

### Procedure

**FIGURE 1:**
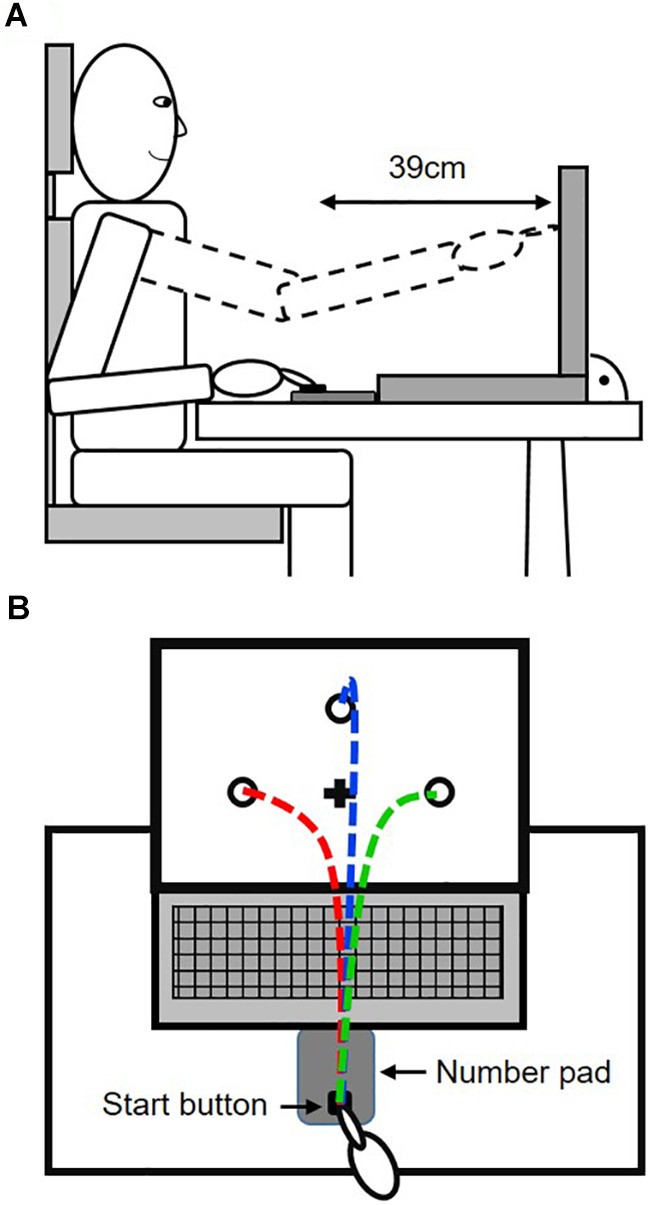
Experimental set-up. **(A)** Side view on the set-up, depicting the reach distance and sitting position of the participants. **(B)** Top view, showing the principal configuration of potential target locations, fixation cross and potential reach trajectories.

In this study, participants had to perform goal-directed reaching movements under target uncertainty during motor decision-making. For that purpose, participants were seated in front of a table, on which a 15″ Laptop (Dell Vostro 3550) and a number pad were placed (see Figure 1A). The number pad was used to spatially control the start position of the fingertip and the reaching distance by defining a start button at the bottom row of the number pad. While this start button was covered by red tape, all other buttons were covered in a black sleeve. Because of using the number pad, movement initiation as the time point of button release could later be exactly defined and used to control the participants’ adherence to the reaction time constraint (see below). A passive reflective marker was attached on top of the fingernail of the right index finger to record fingertip trajectories towards the targets. Fingertip trajectories were recorded at a recording frequency of 150 Hz using a five camera optical motion tracking system (Qualisys Motion Capture Systems, Oqus5, Sweden). The cameras were mounted on a customized frame of 2.60 × 2.70 × 2.70 m in size (width × length × height). The volume covered by all five cameras was approximately 2 × 2 × 2 m (width × length × height), with the participants and the apparatus positioned fully within the covered area. The seating position of the participants was adjusted so that they were able to touch the screen of the laptop without moving the upper body and that fingertip position was always visible for the motion tracking system the at all times during movement execution. The presentation of the targets on the screen was controlled through Presentation^®^ software (Version 17.2, Neurobehavioral Systems, Inc., Berkeley, CA, United States ^1^).

**FIGURE 2:**
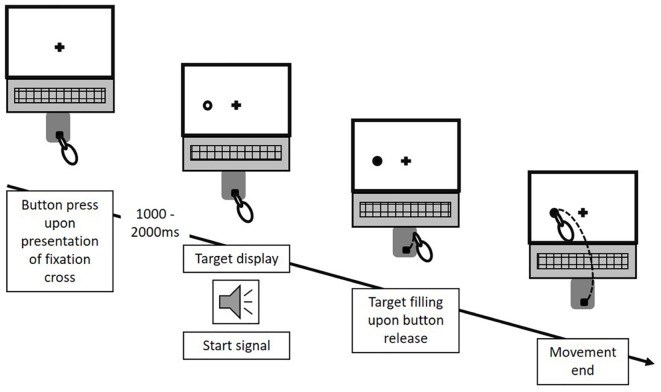
Experimental procedure. In each trial, following a random waiting period of 1–2 s after an initial button press, the potential reach targets were displayed as unfilled circles, appearing at any of the three potential target locations surrounding the fixation cross. Simultaneously, an acoustic start signal triggered participant’s response. Upon button release, the final reach target was indicated through filling of the respective circle. Each trial ended with participants touching a circle on the screen. This figure exemplifies the procedure for one potential trial of Condition A. The same temporal procedure applied for Condition B and C. However, for Condition B and C, two circles were displayed at any of the three location-combinations in each trial.

**FIGURE 3:**
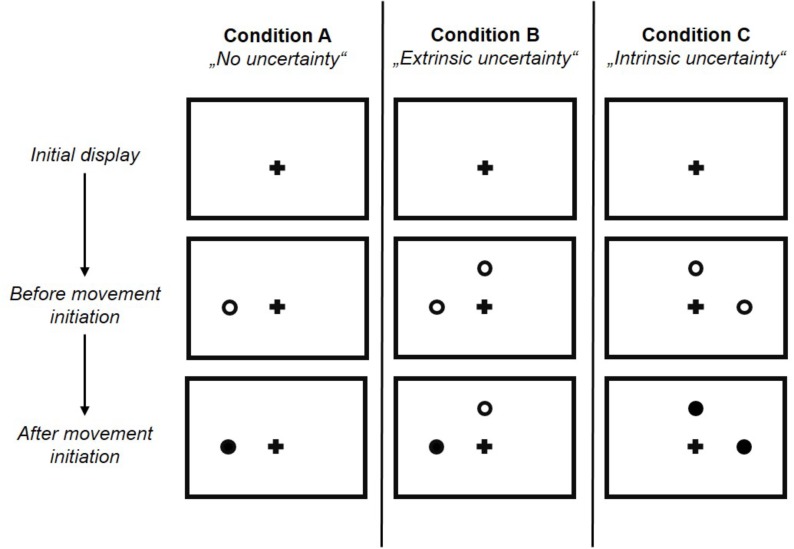
Experimental conditions. Example target displays before and after movement initiation are depicted to highlight the differences between the three experimental conditions. Condition A and B were forced choice conditions with different levels of uncertainty with regard to the amount of available information about the final reach target before movement onset. Condition A (“no uncertainty”) was characterized by very low level of target uncertainty, while Condition B (“extrinsic uncertainty”) was characterized by high level of target uncertainty. In Condition C (“intrinsic uncertainty”) the source of uncertainty was manipulated, i.e., not originating from the limited amount of available target information as in Condition A and B, but from the ambiguity between potential reach targets. Please note that in each of the three experimental conditions, reach targets could be located at any of the three locations and this figure shows different example target locations for each condition.

Target uncertainty during motor decision-making was systematically manipulated across three blocks (i.e., three conditions) of 66 trials each, with the order of conditions being pseudo-randomized across participants. Condition A & B manipulated the level of uncertainty in a forced choice task between low and high, respectively, with Condition A (“no uncertainty”) following a “go-after-you-know”-paradigm, and Condition B (“extrinsic uncertainty”) following a “go-before-you-know”-paradigm (Gallivan et al., 2018). In contrast, the source of uncertainty was altered in Condition C (“intrinsic uncertainty”), originating from the ambiguity of reach targets in a free choice task. All three conditions followed the general procedure as described in Gallivan and Chapman (2014). Participants were visually presented to circular targets (size: 1.3 cm) on the screen, which were located in 7.5 cm distance either above or on the left or right hand side of a fixation cross (i.e., three possible target locations, target size: 1.3 cm, see Figure 1B). At the beginning of each block, participants were informed about the following testing condition and its consequences for the target display through written instructions on the screen. In Condition A, participants were presented to only one circle in each trial, i.e., either on the left, above or on the right of the fixation cross. In contrast, in Condition B and C, participants were always presented to two circles (i.e., three possible combinations of target locations: left-above, left-right, above-right). Each trial started by the participants pressing the start button on the number pad. Subsequently, and depending on the experimental condition, 1–2 unfilled circles were presented at any of the three locations (see Figure 2) following a random waiting period of 1–2 s. Simultaneously, an acoustic start signal sounded and requested participants to initiate their reaching movement within 100–325 ms. Immediately following the release of the start button the final reaching target was indicated through filling of the respective circle. In Condition A (“no uncertainty”) participants were presented to only one target before and after movement onset, so that there was no uncertainty about the reach target during motor decision making (see Figure 3, 1st column). In Condition B (“extrinsic uncertainty”) participants were presented to two targets on the screen, of which only one filled after release of the start button (see Figure 3, 2nd column). Last, in Condition C (“intrinsic uncertainty”) participants had the free choice to which of the two presented unfilled circles they point. Accordingly, both circles filled after movement initiation (see Figure 3, 3rd column). Participants were asked to perform fast and accurate reaching movements from button release to hitting the reach target and to finish the movement within 1 s. Trials that did not meet the reaction time or movement time constraint were excluded from further analysis. In Conditions A and B, each of the three targets were indicated 22 times as the pointing target (i.e., Condition A: 3 targets × 22 trials = 66 test trials; Condition B: 3 targets × 2 possible target combinations × 11 trials = 66 test trials), while in Condition C participants were asked to point about equally often to each of the three targets. Participants were instructed to strictly follow the visual instructions on the screen. Between each block, participants had the chance to rest for a maximum of 5 min to minimize fatigue-induced changes in task performance and motor behavior. Before the start of each block, participants had the chance to familiarize themselves with the task at hand in a practice block consisting of five trials.

### Data Analysis

Data was analyzed using customized Matlab scripts (MATLAB R2011a, Mathworks, Natick, MA, United States). In a first step, to identify endpoints of single reaching movements (i.e., trials) in the continuous data recording of the fingertip marker position in 3D across all trials, local maxima in depth direction were identified separately for each participant and condition. Endpoints were defined as largest position in depth direction with a minimum distance of 34 cm from the start button and within a range of 5 cm. Subsequently, trials were extracted by going backwards 150 sample in time from the sample of the local maxima. Due to the imposed movement time constraint of 1 s, going backwards 150 samples, which were recorded at 150 Hz, was sufficient to extract the complete fingertip trajectories of valid trials. Subsequently, movement velocity was calculated for each trial and sample as the first derivative of the fingertip trajectory with respect to time. Maximum velocity in depth direction (v_max_) was then identified and further used to define movement start and movement end as the first and last sample crossing the threshold of 5% v_max_. Subsequently, *overall* *movement duration* between movement start and end, as well as *deceleration duration*, as the duration between v_max_ and movement end were calculated. To gain insight into the symmetry of the velocity profile, deceleration duration was additionally determined as proportion of overall movement duration in %. Further, *path length* was calculated as the cumulated positional change between samples in horizontal and vertical direction, summed across all samples. Path length in depth direction was not included in this parameter, since the distance between the start button and the screen was fixed by the experimental set-up and could not vary across trials or conditions. For later statistical analyses of experimental condition effects, overall movement duration, deceleration duration and path length of each participant were first averaged across all trials directed towards the same reach target and then averaged across the three targets.

In a next step, to be able to analyze the time course of movement variability, reach trajectories were space-normalized to allow for comparison across reach targets and conditions. Space-normalization was preferred over normalizing the trajectories in time, as we assumed differences in movement duration between experimental conditions, which potentially would have affected later analysis [for a more detailed discussion on this issue, see Gallivan and Chapman (2014)]. To illustrate one relevant potential issue related to time normalization, assume unconstrained reaching movements under low target uncertainty being characterized by bell-shaped velocity profiles with equal amount of time spent for acceleration and deceleration of the fingertip. Empirical evidence suggests that an experimentally induced increase in reach target uncertainty results in an increase in overall movement duration (Gallivan and Chapman, 2014). This increase could in principal result from an increase in (A) only acceleration duration, (B) only deceleration duration, or (C) both symmetrically. If (A) or (B) would prove to be true, comparing time normalized reach trajectories performed under low and high target uncertainty would result in the comparison of data samples from different phases (i.e., acceleration and deceleration phase). Because of the hypothesized different contribution of both phases to the control of voluntary movements (Woodworth, 1899; Elliott et al., 2001; Elliott et al., 2010), we aimed for normalizing the reach trajectories to a dimension that did not differ between experimental conditions, namely, the distance between movement start and end, to avoid potential artifacts in the outcome of the data analysis. Each trial was normalized to 11 equidistant samples between movement start and movement end. Consequently, each sample corresponds to 10% of the traveled distance in depth direction (i.e., between the start button and laptop screen) starting from 0% (1st sample). Reducing the sample resolution with respect to important kinematic events (e.g., peak velocity, peak acceleration) or certain percentages of movement distance or time is a standard approach in motor control research, especially with regard to the analysis of movement variability across the time course of movement execution (e.g., Scholz and Schöner, 1999; Cuijpers et al., 2004; Krüger et al., 2011; van der Steen and Bongers, 2011). Subsequently, variability in fingertip position was calculated following the procedure of previous work from our group (Krüger et al., 2011) as the within-subject between-trial standard deviation of the mean horizontal fingertip position, separately for each participant, condition, target position and each of the 11 samples. Following that, fingertip variability was averaged across the three reach targets.

### Statistical Analysis

Statistical analysis was calculated using SPSS Statistics 23 (IBM Corp., Armonk, NY, United States). Differences in overall movement duration, acceleration duration, deceleration duration, and path length between experimental conditions were analyzed using repeated measurement ANOVA with Condition as within-subject factor. The time course of variability of fingertip position was analyzed using repeated measurement ANOVA with Condition as within-subject factor and time sample as repeated factor. In addition, endpoint variability was analyzed as the variability of fingertip position at the 11th sample by using repeated measurement ANOVA with Condition as within-subject factor. Post-hoc comparisons were calculated using paired-sample *t*-test to further investigate significant differences between Conditions, and one-way ANOVA for further analyses of significant differences between samples. The critical level of statistical significance was set to α ≤ 0.05. Greenhouse-Geisser corrections of the degrees of freedom were applied if the assumption of sphericity for the ANOVA was violated. Partial eta-square (*η_p_^*2*^*) was calculated to aid in the interpretation of the magnitude of observed effects. In accordance with the recommendation of Sink and Stroh (2006) *η_p_^*2*^* ≥ 0.06 was considered as medium effect and *η_p_^*2*^* ≥ 0.14 as large effect.

## Results

**FIGURE 4:**
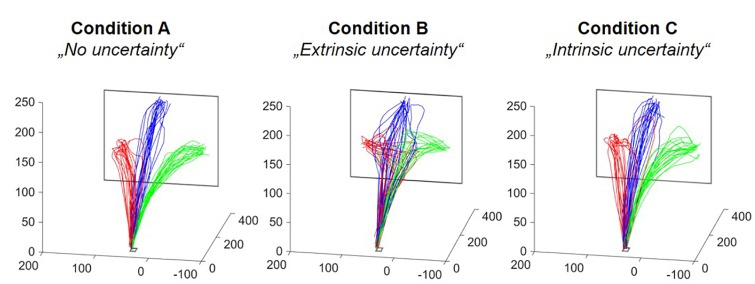
Reach trajectories of one representative participant. Each subfigure depicts all reach trajectories executed in one of the three experimental conditions. The different colors relate to the different final reach targets. The axes represent the three dimension in space plotted in mm. The differences in between-trial variability of fingertip trajectories become clearly visible.

Qualitatively, the different levels and sources of target uncertainty had a clear influence on reaching movements (see Figure 4). These differences could also be supported by the outcomes of the statistical data analyses, which will be described in the following. Reported absolute values for the different experimental conditions refer to the mean ± SE.

### Spatial and Temporal Movement Characteristics

**FIGURE 5:**
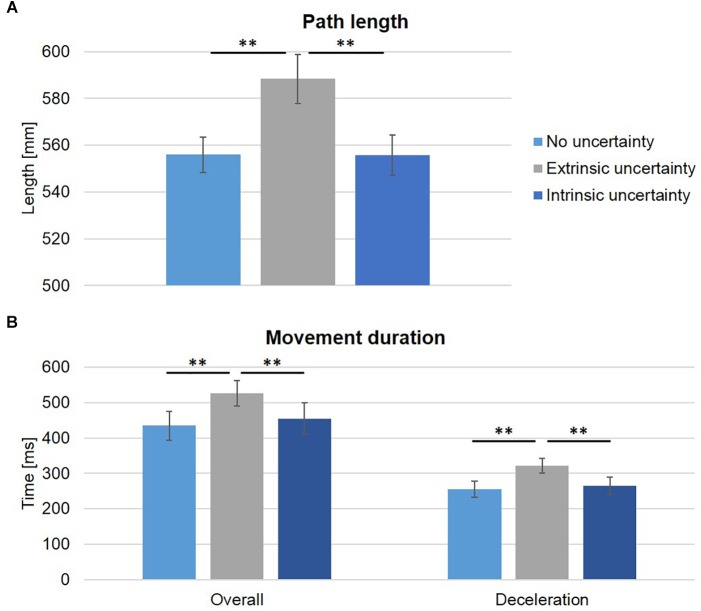
Spatial and temporal movement characteristics. **(A)** The group averages (±SE) of path length (horizontal and vertical direction, in mm) are displayed for the three experimental conditions. Path length was significantly increased under extrinsic uncertainty as compared to the other two conditions. **(B)** Average overall movement duration and duration of deceleration are displayed. Again, the mean ± SE are plotted. Overall movement duration was significantly increased under extrinsic uncertainty, which was related to the significantly increased deceleration duration in this condition. Note that although the proportion of time spent after peak velocity was not statistically different between experimental conditions; ^∗∗^*p* < 0.01.

Target uncertainty had a significant influence on temporal and spatial movement characteristics. Path length was significantly increased under extrinsic target uncertainty (Condition B: 588.28 ± 10 mm, vs. A: 553.90 ± 8 mm, and C: 555.83 ± 9 mm, see Figure 5A), as indicated by a main effect of condition (*F*(2,18) = 16.26, *p* < 0.001, *η_p_^*2*^* = 0.64) and subsequent post-hoc comparisons (A vs. B: *t*(9) = -5.03, *p* = 0.001; A vs. C: *t*(9) = -0.30, *p* > 0.05; B vs. C: *t*(9) = 4.58, *p* < 0.001). Similarly, overall movement duration was significantly longer under extrinsic uncertainty (Condition B: 562 ± 112 ms, see Figure 5B) as compared to intrinsic or no target uncertainty (Condition C: 455 ± 142 ms and Condition A: 434 ± 129 ms, respectively) which did not differ from each other, as indicated by a significant main effect of condition (*F*(2,18) = 13.74, *p* < 0.001, *η_p_^*2*^* = 0.60) and subsequent pairwise comparisons (A vs. B: *t(*9) = -5.41, *p* < 0.001; A vs. C: *t*(9) = -1.06, *p* > 0.05; B vs. C: *t*(9) = 3.75, *p* = 0.005). The differences in overall movement duration could be attributed to a significantly longer deceleration duration under extrinsic target uncertainty as compared to the two other experimental conditions (Condition A: 255 ± 71 ms, B: 322 ± 65 ms, C: 265 ± 78 ms, see Figure 5B) as indicated by a significant main effect of condition (*F*(2,18) = 12.39, *p* < 0.001, *η_p_^*2*^* = 0.58) and post-hoc pairwise comparisons (A vs. B: *t*(9) = -3.84, *p* = 0.004; A vs. C: *t*(9) = -0.73, *p* > 0.05; B vs. C: *t*(9) = 5.07, *p* = 0.001). The absolute values of deceleration duration represented 59.02%, 61.67%, and 58.93% of overall movement duration for Condition A, B, and C, respectively. While the absolute amount of time spent after peak velocity was significantly different between the three experimental conditions, the proportion of time was not (*p* > 0.05).

### Movement **V**ariability

**FIGURE 6:**
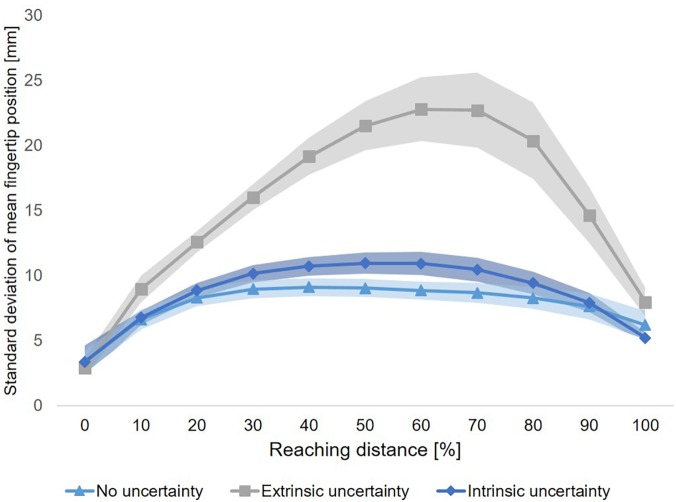
Time course of variability of fingertip position. For each of the three experimental conditions, the time course of movement variability (mean ± SE) is displayed. Each of the three time courses show an increase-decrease pattern of movement variability, which is most strongly pronounced for Condition B (“extrinsic uncertainty”). There is no difference in variability of fingertip position at movement start between the three experimental conditions.

Variability of fingertip position across the time course of movement execution showed a clear increase-decrease pattern for all conditions. This qualitative observation was supported by a significant main effect of Sample (*F*(10,90) = 26,60, *p* < 0.001, *η_p_^*2*^* = 0.75). Importantly, the time course of variability of fingertip position also showed clear differences between experimental conditions (see Figure 6). This qualitative observation was supported by a significant main effect of Condition across all samples (*F*(2,18) = 28.39, *p* < 0.001, *η_p_^*2*^* = 0.76) and at movement end (*F*(2,18) = 3.85, *p* = 0.04, *η_p_^*2*^* = 0.30). In addition, the interaction of Condition × Sample was significant (*F*(20,180) = 18.80, *p* < 0.001, *η_p_^*2*^* = 0.60). Post-hoc comparisons to further elucidate the differences in variability of fingertip position across the time course of movement execution revealed a graded pattern. First, fingertip trajectories in Condition A (“no uncertainty”) showed a generally lower variability as compared to Condition B (“extrinsic uncertainty”), as indicated by a significant main effect of Condition (*F*(1,9) = 36.24, *p* < 0.001, *η_p_^*2*^* = 0.80). This difference became evident especially shortly after movement start until movement end (see Figure 6), as indicated by a significant interaction of Condition × Sample (*F*(10,90) = 21.61, *p* < 0.001, *η_p_^*2*^* = 0.71) and post-hoc comparisons of single samples (see Table 1).

**Table 1:** Statistical parameters regarding the analysis of the time course of variability of fingertip position for the three experimental conditions.

Time course	Condition A vs. B	Condition A vs. C	Condition B vs. C
Movement start	n.s.	n.s.	n.s.
10%	n.s.	n.s.	*t*(9) = 2.40, *p* = 0.04
20%	*t*(9) = –3.79, *p* = 0.004	n.s.	*t*(9) = 3.80, *p* = 0.04
30%	*t*(9) = –5.01, *p* = 0.001	*t*(9) = –2.55, *p* = 0.03	*t*(9) = 4.54, *p* = 0.001
40%	*t*(9) = –6.04, *p* < 0.001	*t*(9) = –2.46, *p* = 0.04	*t*(9) = 4.94, *p* = 0.001
50%	*t*(9) = –6.63, *p* < 0.001	*t*(9) = –2.35, *p* = 0.04	*t*(9) = 5.10, *p* = 0.001
60%	*t*(9) = –6.34, *p* < 0.001	*t*(9) = –2.29, *p* = 0.05	*t*(9) = 4.92, *p* = 0.001
70%	*t*(9) = –5.91, *p* < 0.001	n.s.	*t*(9) = 4.51, *p* = 0.001
80%	*t*(9) = –5.25, *p* = 0.001	n.s.	*t*(9) = 4.14, *p* = 0.003
90%	*t*(9) = –5.13, *p* = 0.001	n.s.	*t*(9) = 3.58, *p* = 0.006
Movement end	*t*(9) = –2.89, *p* = 0.02	n.s.	*t*(9) = 3.16, *p* = 0.01


*For clarity reasons, only statistical parameters of significant differences are displayed, with the significance level at *α* ≤ 0.05. Each line corresponds to the pairwise comparison of variability at one sample, which equals 10% of the traveled reach distance.*

The time courses of movement variability also showed significant differences between the experimental conditions with different sources of target uncertainty during motor decision-making. Specifically, fingertip trajectories showed lower variability early after movement start until movement end in the case of intrinsic uncertainty as compared to extrinsic target uncertainty (Condition C vs. B, respectively, see Figure 6 and Table 1). This observation was supported by a significant main effect of Condition (*F*(1,9) = 26.44, *p* = 0.001, *η_p_^*2*^* = 0.75), a significant interaction of Condition × Sample (*F*(10,90) = 10.64, *p* < 0.001, *η_p_^*2*^* = 0.54) and post-hoc comparisons of single samples (see Table 1). Interestingly, significant differences in variability of fingertip position also became evident between Condition A and C, especially during the mid of movement execution, but not at movement start or end (see Figure 6), as revealed by a significant interaction of Condition × Sample (*F*(10,90) = 2.57, *p* = 0.01, *η_p_^*2*^* = 0.22) and subsequent post-hoc comparisons of single samples (see Table 1).

## Discussion

In this study, healthy young adults performed reaching movements under three different conditions of target uncertainty. The aim was to investigate the influence of different levels and sources of target uncertainty during motor decision-making on movement execution. To quantify the effect of target uncertainty, variability of fingertip position during the time course of movement execution and at movement end was analyzed, in addition to temporal and spatial movement characteristics. Overall, the results of the study suggest that the time course analysis of movement variability can reveal the effect of different sources of target uncertainty on the processing of potential action plans during motor decision making, which are not captured with standard temporal and spatial kinematic analyses.

### Influence of Different Levels of Uncertainty

The first main outcome of our study is that different levels of extrinsic target uncertainty directly affect temporal and spatial movement characteristics of goal-directed reaching movements. This supports existing empirical evidence, which has been accumulated in the last recent years (e.g., Trommershäuser et al., 2005; Song and Nakayama, 2009; Gallivan et al., 2011; Gallivan and Chapman, 2014). During that time, the theoretical approach to movement planning has changed from a hierarchical system, assuming a serial process of action planning, to a theory of parallel action planning (Cisek, 2007; Cisek and Kalaska, 2010). The basic assumption is that the motor system, to account for uncertainties and dynamics in the environment, specifies and prepares multiple potential actions in parallel, of which one is finally selected. More recently, Cisek and Pastor-Bernier (2014) proposed that these two processes, action specification and selection even go in parallel with action execution – termed “embodied decision making”. Empirical evidence stemming from neurophysiological and kinematic data seems to support this view. Work by e.g., the group of Chapman, Gallivan and colleagues provided empirical evidence that, in the presence of target uncertainty, multiple potential action plans are prepared in parallel and that action planning is influenced by e.g., the spatial distribution of targets or their likelihood of appearance (Chapman et al., 2010; Gallivan et al., 2011; Gallivan and Chapman, 2014). They analyzed spatial and temporal characteristics of the movement trajectories (e.g., movement duration, path length, etc.) to highlight differences in movement execution between conditions with low or high target uncertainty due to the availability of information about the final reach target.

In our study, we were able to replicate these findings. We found a significant increase in overall movement duration in the presence of high as compared to no extrinsic uncertainty about the final reach target (Condition B vs. A, respectively). This increase could be attributed to a significantly longer deceleration duration in that condition, which suggests greater amount of online correction processes taking place in the presence of high extrinsic target uncertainty (Elliott et al., 2001; Elliott et al., 2010). However, the proportion of time spent after peak velocity was statistically similar between groups, which limits the general validity of the previous suggestion. In all three conditions, about 60% of overall movement time was spent after peak velocity, indicating a general asymmetry in the velocity profile with longer times spent for deceleration in all conditions. This finding, in combination with the significantly longer absolute time spent after peak velocity, suggests that, under high extrinsic uncertainty, both acceleration and deceleration duration are increased as compared to no uncertainty, with only deceleration duration reaching the statistical level of significance. In the existing literature, increased movement duration under higher levels of extrinsic target uncertainty are explained as resulting from the simultaneous increase in path length, as also observed in our study (see Figure 4), reflecting greater lateral deviation from a straight path between movement start and endpoint (Gallivan and Chapman, 2014). This finding is commonly discussed as resulting from a competition between two different movement plans (for reaching to either one or the other target). Because of this competition, trajectories are initially directed towards a midpoint between the two potential targets, and only after the final reaching target is known, redirected towards it (Gallivan and Chapman, 2014; Gallivan et al., 2018). Alternatively, this finding is discussed as reflecting the execution of a movement plan that optimizes costs for later motor corrections (Nashed et al., 2017; Gallivan et al., 2018). The analyzed spatial and temporal movement parameters in our study do not allow any conclusion in favor or against any of the two options.

Similarly, fingertip variability during the time course of movement execution was by far the highest when motor decision-making took place under high level of extrinsic target uncertainty (Condition B) as compared to the other two conditions. This is a striking evidence for the impact of different levels of extrinsic target uncertainty during motor decision-making on movement execution. It also reflects the dynamics of the motor decision-making process in case of high target uncertainty (Condition B). Even in trials with similar environmental conditions, i.e., with regard to the location of potential reach targets or the onset of the final target display, the competition between multiple potential action plans varied across trials, directly affecting the finally performed movement path, and the variability between movement paths across trials. Overall, within-subject between-trial variability of fingertip position showed an increase-decrease pattern across the time course of movement execution, with low variability at movement end (∼5–10 mm from mean endpoint, see Figure 6). This pattern is similar to previous studies of our group analyzing movement variability to gain insight into movement planning and control processes (see e.g., Krüger et al., 2011, 2012) and illustrates the effectiveness of online-control mechanisms.

### Effect of Different Sources of Uncertainty

The second main outcome of our study is that not only the level of target uncertainty affects the parallel processing of multiple potential action plans during motor decision-making, but also the source of target uncertainty and that this can be revealed through analyzing the time course of movement variability. Lipshitz and Strauss (1997) highlighted the existence of different types of uncertainty, which can be classified e.g., according to their source. Following their proposition, decision uncertainty can originate from the limited amount of information about the final reach target (“extrinsic uncertainty” in our study) as well as from the ambiguity of reach options between which participants can freely choose (“intrinsic uncertainty” in our study). Manipulating the amount of information about the final reach target is a common experimental procedure in motor decision making-research (see Gallivan et al., 2018 for a review) and also used in our study to imply conditions of no and high level of extrinsic target uncertainty (Condition A and B, respectively). Implying different sources of uncertainty are much less common experimental manipulations, yet. So far, conditions of free choice are commonly used to reveal and manipulate individual preferences of choice options [see Gallivan and Chapman (2014) for a short summary on these results].

In our study, target preference should not have been a relevant aspect in the free choice condition (Condition C, “intrinsic uncertainty”), as the potential reach targets were not associated with any kind of reward or penalty. In contrast, participants were instructed to reach about equally often to each of the three targets across all trials. This allowed us to focus on the different source of decision uncertainty in this condition as compared to the other two experimental conditions and its consequences on the process of motor decision-making. In the two forced choice conditions (Condition A and B) the motor decision-making strategies were externally imposed by the time point of indication of the final reach target. In Condition A, where the final reach target was cued immediately with stimulus onset, decision uncertainty was minimal, thus, allowed participants to straightly reach towards the indicated target. In contrast, in Condition B, where the final reach target was cued only after movement onset, the experimental set-up enforced the ongoing parallel processing of multiple potential action plans during movement execution. This “embodied decision-making” strategy is supposed to be beneficial to cope with uncertainties and environmental dynamics during movement execution (Cisek and Pastor-Bernier, 2014). The same strategy would also allow to successfully cope with the intrinsic target uncertainty in the free choice condition (Condition C), which should then reflect in the movement kinematics of the reach trajectories. However, when participants were allowed to freely choose between potential reach targets, an alternative motor decision-making strategy of serial action planning could have also been applied. To minimize reach target uncertainty at the time point of movement start, participants could have decided for any of the two potential reach targets immediately after stimulus onset, which would have allowed them to reach straightly to the chosen target, similarly to Condition A, where the reach target was cued before movement onset.

The analysis of spatial and temporal movement characteristics revealed significant differences in path length and overall movement duration between intrinsic and extrinsic target uncertainty (Condition C and B, respectively), but not between the intrinsic and no uncertainty condition (Condition A). This finding seems to support the assumption that, under free choice conditions, decisions on the final reach target are made using a strategy that minimizes uncertainty at movement start. However, the analysis of movement variability revealed a distinct pattern. In contrast to the findings in spatial and temporal movement characteristics, the time course analysis of fingertip variability revealed significant differences between reaching movements under extrinsic (Condition A and B) and intrinsic target uncertainty (Condition C). It became evident that under intrinsic uncertainty (i.e., target ambiguity during free choice) fingertip variability was higher than under low extrinsic target uncertainty early after movement onset until the last quarter of the reach trajectory. This suggests that competition between action plans related to reaching towards different potential targets was still ongoing during movement execution and not finalized at movement onset and supports the theory of embodied decision making (Cisek and Pastor-Bernier, 2014). The results are also compatible with attention based models of selective reaching (Tipper et al., 1997, 1998; Welsh et al., 1999; Welsh and Elliott, 2004). In these models, it is hypothesized that the presence of a non-target stimulus in the environment automatically evokes a neural response, which has to be inhibited to successfully reach towards the target stimulus. This inhibition process acts as a distractor on the initiation and execution of the actual reach movement. From this perspective, the pure presentation of the second potential reach target in the free choice condition (Condition C) could have affected the time course of movement variability by interfering with the preparation and execution of the reaching movement towards the selected target. Thus, even if response selection in the intrinsic uncertainty condition (Condition C) would have been finished before movement initiation, the response inhibition process of the non-selected reach target could have affected the kinematics towards the selected reach target. In general, all of the three above mentioned models (Tipper et al., 1998; Welsh and Elliott, 2004; Cisek and Pastor-Bernier, 2014) agree in their basic assumption that the presence of multiple potential reach targets in the environment automatically evoke parallel responses that compete against each other. At the present moment, we cannot finally conclude whether the observed differences in the time course of movement variability result from the ongoing decision process between potential action plans, as proposed by Cisek and Pastor-Bernier (2014), or from the inhibition process of the non-selected reach target, as proposed by Tipper et al. (1998) and Welsh and Elliott (2004). Further research will be necessary to clarify this point.

Overall, the findings suggests that the time course analysis of movement variability of the end-effector can reveal dynamics in the motor decision-making process, which cannot be captured by standard kinematic movement analyses. The observed differences in the time course of fingertip variability between the conditions of intrinsic uncertainty (Condition C) and low level of extrinsic uncertainty (Condition A) are much smaller as between those two conditions and Condition B (“extrinsic uncertainty”, see Figure 6). This might suggest different levels of uncertainty between the experimental conditions and highlights the relevance of accounting for the different sources *and* levels of uncertainty in future studies on that topic.

### Methodological Considerations

To the best of our knowledge, this is the first study investigating the effect of different sources of target uncertainty on reach kinematics. On this basis, we are aware that it does not take sufficient account for all critical points, which need further consideration in future research. First, we were able to show differences in end-effector variability related to different sources of uncertainty. It is intriguing to conclude that a higher amount of movement variability during movement execution directly relates to a higher level of uncertainty during motor decision-making. However, in the current study we cannot exactly determine the level of uncertainty for the free choice condition. Further studies investigating the effect of different levels of uncertainty in the presence of target ambiguity (“intrinsic uncertainty”), or comparing the effect of similar levels of uncertainty between different sources of uncertainty are needed to further elucidate that point. Second, in this study we analyzed the time course of fingertip variability during movement execution, providing information about end-effector movement control process. However, sophisticated mathematical approaches have been developed, which allow gaining insight into the coordination of the abundant effector degrees of freedom that underlies the control of fingertip position (e.g., Scholz and Schöner, 1999; Müller and Sternad, 2004; Krüger et al., 2017). The application of these approaches might prove to be valuable for further progress in integrating empirical evidence on movement planning and control with psychological theories and computational models on decision-making. Last, we acknowledge the existence of different methodological approaches in calculating movement variability and its changes over time, in particular with regard to (1) time- vs. space-normalization of the movement trajectories and (2) reducing the time resolution to relevant events vs. functional comparison (FDA). For the trajectory normalization we provided our rationale – to normalize to the dimension that varies the least between conditions (cf., Gallivan and Chapman, 2014). While space-normalization is less common in the existing literature, we are convinced by its adequacy for our current study. With regard to the second critical methodological decision, we followed the common procedure in motor control research, without having any reason for considering FDA as more or less appropriate for the purpose of our study. Future studies with a stronger methodological focus might target this aspect.

## Conclusion

In this study, we investigated the effect of different levels and sources of target uncertainty during motor decision making on the kinematics of reaching movements. In line with previous research, we found increased path length, overall movement duration and deceleration duration with increasing level of extrinsic target uncertainty. Similarly, we found differences in the time course of within-subject, across-trial fingertip variability between different levels of extrinsic target uncertainty, with higher amount of variability going along with higher level of uncertainty. Importantly, we also found increased variability of fingertip position during the time course of movement execution in the presence of intrinsic uncertainty as compared to low level of extrinsic uncertainty, but no differences in path length or movement duration. This suggests that under intrinsic uncertainty, i.e., target ambiguity in free choice condition, multiple potential actions are planned and compete for action during movement execution. This is a remarkable finding, since under the condition of free choice, as tested in this study, in principal a motor decision-making strategy of serial action planning could have been applied to minimize decision uncertainty before movement onset. However, the time course analysis of movement variability revealed that the motor decision-making process was still ongoing during movement execution. Importantly, these differences were not captured by standard kinematic movement analyses. In conclusion, during motor decision making under intrinsic target uncertainty, the strategy of ongoing parallel processing of multiple potential actions during movement execution that allows coping with uncertainties and environmental dynamics seems to be favored over a strategy of serial action planning that minimizes decision uncertainty before movement onset.

## Data Availability Statement

The raw data supporting the conclusions of this manuscript will be made available by the authors, without undue reservation, to any qualified researcher.

## Author Contributions

Both authors of this study contributed substantially to its conception, the interpretation of data, as well as drafting and revising earlier versions of the manuscript and also approved the version to be published and agreed to be accountable for all aspects of the work. MK was responsible for data acquisition and analysis.

## Conflict of Interest Statement

The authors declare that the research was conducted in the absence of any commercial or financial relationships that could be construed as a potential conflict of interest.

## Footnotes

1 www.neurobs.com
