# Corrigendum: Target Uncertainty During Motor Decision-Making: The Time Course of Movement Variability Reveals the Effect of Different Sources of Uncertainty on the Control of Reaching Movements

**DOI:** 10.3389/fpsyg.2019.00913

**Published:** 2019-04-24

**Authors:** Melanie Krüger, Joachim Hermsdörfer

**Affiliations:** Chair of Human Movement Science, TUM Department of Sport and Health Sciences, Technical University of Munich, Munich, Germany

**Keywords:** reaching movements, sensorimotor control, movement planning, motor control, embodied decision making, time course of variability, kinematics

In the original article, there was a mistake in Figure 2 and the corresponding figure legend as published. The mistake relates to an incorrect description of the timeline of stimulus presentation and the occurrence of the start signal. While it was stated, that the start signal occurred 1,000–2,000 ms after target display, both actually occurred at the exact same time. The correct Figure 2 and legend appears below.

**Figure 2:**
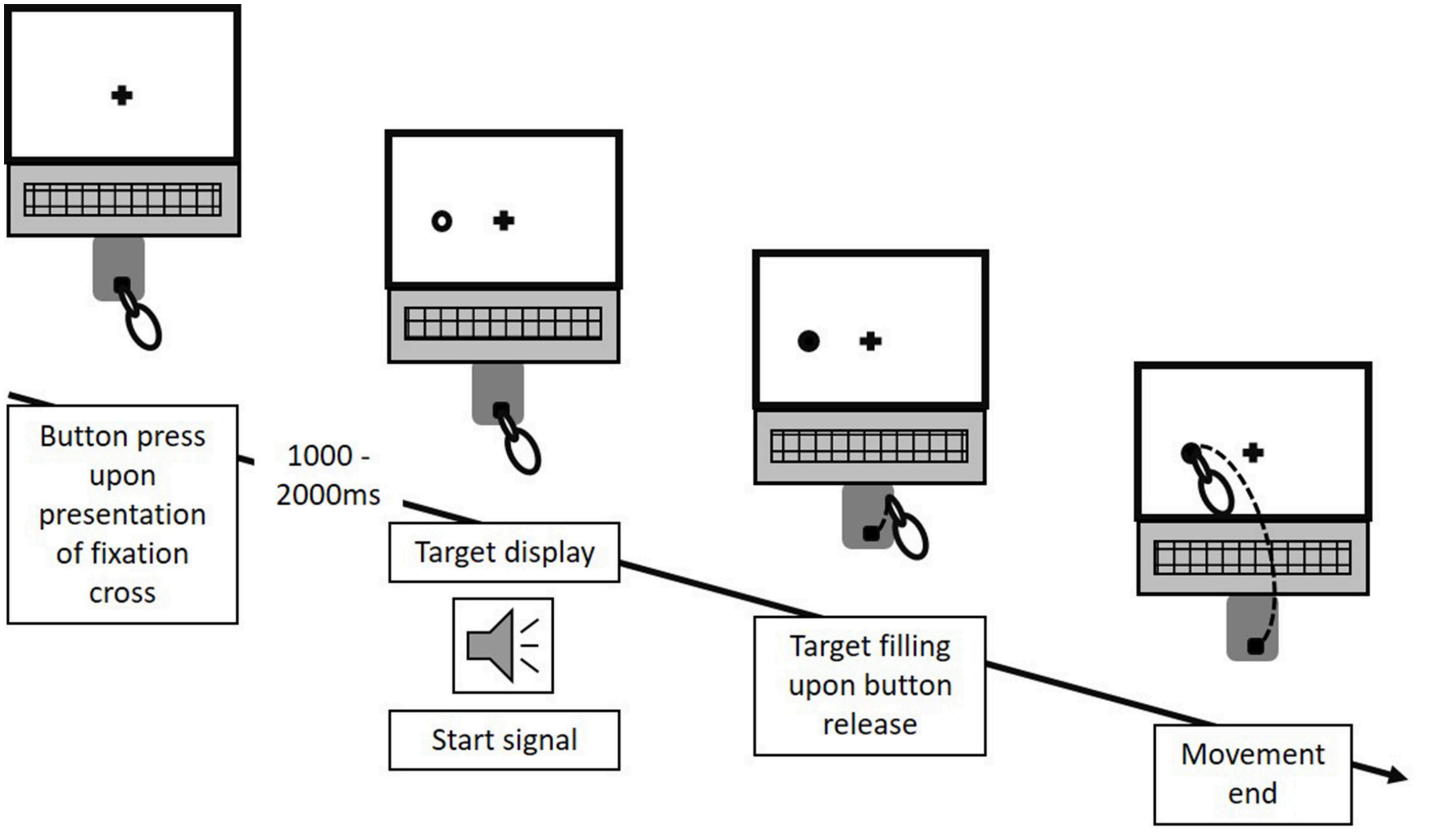
Experimental procedure. In each trial, following a random waiting period of 1–2 s after an initial button press, the potential reach targets were displayed as unfilled circles, appearing at any of the three potential target locations surrounding the fixation cross. Simultaneously, an acoustic start signal triggered participant's response. Upon button release, the final reach target was indicated through filling of the respective circle. Each trial ended with participants touching a circle on the screen. This figure exemplifies the procedure for one potential trial of Condition A. The same temporal procedure applied for Condition B and C. However, for Condition B and C, two circles were displayed at any of the three location-combinations in each trial.

Due to the error in the description of the timeline of stimulus presentation and the occurrence of the start signal described above, a correction has been made to the **Methods**, subsection **Procedure**, paragraph two:

“Target uncertainty during motor decision-making was systematically manipulated across three blocks (i.e., three conditions) of 66 trials each, with the order of conditions being pseudo-randomized across participants. Condition A & B manipulated the level of uncertainty in a forced choice task between low and high, respectively, with Condition A (“no uncertainty”) following a “go-after-you-know”-paradigm, and Condition B (“extrinsic uncertainty”) following a “go-before-you-know”-paradigm (Gallivan et al., 2018). In contrast, the source of uncertainty was altered in Condition C (“intrinsic uncertainty”), originating from the ambiguity of reach targets in a free choice task. All three conditions followed the general procedure as described in Gallivan and Chapman (2014). Participants were visually presented to circular targets (size: 1.3 cm) on the screen, which were located in 7.5 cm distance either above or on the left or right hand side of a fixation cross (i.e., three possible target locations, target size: 1.3 cm, see Figure 1B). At the beginning of each block, participants were informed about the following testing condition and its consequences for the target display through written instructions on the screen. In Condition A, participants were presented to only one circle in each trial, i.e., either on the left, above or on the right of the fixation cross. In contrast, in Condition B and C, participants were always presented to two circles (i.e., three possible combinations of target locations: left-above, left-right, above-right). Each trial started by the participants pressing the start button on the number pad. Subsequently, and depending on the experimental condition, 1–2 unfilled circles were presented at any of the three locations (see Figure 2) following a random waiting period of 1–2 s. Simultaneously, an acoustic start signal sounded and requested participants to initiate their reaching movement within 100–325 ms. Immediately following the release of the start button the final reaching target was indicated through filling of the respective circle. In Condition A (“no uncertainty”) participants were presented to only one target before and after movement onset, so that there was no uncertainty about the reach target during motor decision making (see Figure 3, 1st column). In Condition B (“extrinsic uncertainty”) participants were presented to two targets on the screen, of which only one filled after release of the start button (see Figure 3, 2nd column). Last, in Condition C (“intrinsic uncertainty”) participants had the free choice to which of the two presented unfilled circles they point. Accordingly, both circles filled after movement initiation (see Figure 3, 3rd column). Participants were asked to perform fast and accurate reaching movements from button release to hitting the reach target and to finish the movement within 1 s. Trials that did not meet the reaction time or movement time constraint were excluded from further analysis. In Conditions A and B, each of the three targets were indicated 22 times as the pointing target (i.e., Condition A: 3 targets × 22 trials = 66 test trials; Condition B: 3 targets × 2 possible target combinations × 11 trials = 66 test trials), while in Condition C participants were asked to point about equally often to each of the three targets. Participants were instructed to strictly follow the visual instructions on the screen. Between each block, participants had the chance to rest for a maximum of 5 min to minimize fatigue-induced changes in task performance and motor behavior. Before the start of each block, participants had the chance to familiarize themselves with the task at hand in a practice block consisting of five trials.”

In addition, there was an error in the summary of the results on endpoint variability in the Discussion. It was stated that endpoint variability was equal at movement end for all three conditions. In fact, as correctly reported in Table 1 and the Results, endpoint variability was significantly higher for Condition B.

A correction has been made to the **Discussion**, subsection **Influence of Different Levels of Uncertainty**, paragraph three:

“Similarly, fingertip variability during the time course of movement execution was by far the highest when motor decision-making took place under high level of extrinsic target uncertainty (Condition B) as compared to the other two conditions. This is a striking evidence for the impact of different levels of extrinsic target uncertainty during motor decision-making on movement execution. It also reflects the dynamics of the motor decision-making process in case of high target uncertainty (Condition B). Even in trials with similar environmental conditions, i.e., with regard to the location of potential reach targets or the onset of the final target display, the competition between multiple potential action plans varied across trials, directly affecting the finally performed movement path, and the variability between movement paths across trials. Overall, within-subject between-trial variability of fingertip position showed an increase-decrease pattern across the time course of movement execution, with low variability at movement end (~5–10 mm from mean endpoint, see Figure 6). This pattern is similar to previous studies of our group analyzing movement variability to gain insight into movement planning and control processes (see e.g., Krüger et al., 2011, 2012) and illustrates the effectiveness of online-control mechanisms.”

The authors apologize for these errors and state that they do not change the scientific conclusions of the article in any way. The original article has been updated.
